# Safety and Efficacy of Orally Administered SJP-0008 in Central Retinal Artery Occlusion: A Phase IIa Randomized Clinical Trial

**DOI:** 10.1016/j.xops.2025.100965

**Published:** 2025-10-10

**Authors:** Satoru Tsuda, Hiroshi Kunikata, Kazuki Hashimoto, Toshifumi Asano, Azusa Ito, Mitsuhide Yoshida, Masayuki Yasuda, Fumihiko Nitta, Toru Nakazawa

**Affiliations:** 1 Department of Ophthalmology, Tohoku University Graduate School of Medicine, Sendai, Miyagi, Japan; 2 Division of Ophthalmic Precision Medicine Development, United Centers for Advanced Research and Translational Medicine (ART), Tohoku University Graduate School of Medicine, Sendai, Miyagi, Japan; 3 Asano Ophthalmology Clinic, Shiroishi, Miyagi, Japan; 4 Department of Ophthalmology, Sendai City Hospital, Sendai, Miyagi, Japan

**Keywords:** Calpain inhibitors, Central retinal artery occlusion, ETDRS, Visual acuity

## Abstract

**Purpose:**

To assess the efficacy and safety of orally administered calpain inhibitor SJP-0008 in Japanese patients with central retinal artery occlusion (CRAO), to establish a disease registry for the prospective tracking of observational data from patients with CRAO, intended for regulatory use, and to support the development of new therapeutic agents for CRAO.

**Design:**

This was a 2-part study. Part 1 was a physician-/investigator-initiated, phase IIa, single-center, randomized, double-blinded, parallel-group study. Part 2 was a prospective cohort study, during which the CRAO registry was established, and included patients diagnosed with CRAO (including a nonrandomized registry cohort, the non-SJP group, which did not receive SJP-0008). Additionally, in part 2, a combined analysis was performed using data from part 1 patients.

**Participants:**

The study participants were patients recruited within 48 hours of developing CRAO.

**Methods:**

SJP-0008 administration was initiated at least 3 hours but no more than 48 hours after the onset of CRAO. Patients were randomized in a 1:1 ratio using masked randomization to receive either 100-mg or 200-mg doses of SJP-0008. The dosing period was defined as the 4-week postinitiation period (up to 29 days), followed by an 8-week postobservation phase.

**Main Outcome Measures:**

The main outcome measure was to determine the efficacy of SJP-0008 treatment; the primary endpoint was the change in ETDRS visual acuity at 12 weeks in the target eye of patients with CRAO.

**Results:**

The study included 28 patients (mean age: 68.8 ± 14.9 years; 78.6% male). ETDRS scores (mean [95% confidence interval]) were higher at week 12 than at baseline in both the 100-mg (27.9 letters [10.14, 45.61]) and 200-mg (25.7 letters [12.02, 39.40]) SJP-0008 groups, in contrast to the non-SJP group (10.2 letters [4.58, 15.76]). The improvement in the 200-mg SJP-0008 group was greater than in the nonrandomized non-SJP group (*P* = 0.040). No safety concerns were identified.

**Conclusions:**

The preliminary study supports the safety and efficacy of oral administration of SJP-0008 for treating CRAO, with greater improvement compared with the nonrandomized registry cohort. However, large-scale, multicenter randomized controlled trials are warranted to validate the findings of this study.

**Financial Disclosure(s):**

Proprietary or commercial disclosure may be found in the Footnotes and Disclosures at the end of this article.

Central retinal artery occlusion (CRAO) causes acute vision loss and is characterized by thromboembolic occlusion of the central retinal artery and its branches.^1^^,^^2^ The overall incidence of CRAO is reported to be 1 in 100 000 persons and 1 in 10 000 among outpatients visiting ophthalmologists.^3^ In Japan, the age-standardized incidence rate of CRAO is 2.53 per 100 000 person-years.^4^ There are no approved therapies for CRAO. Treatments such as vasodilators, medications to reduce intraocular pressure, ocular massage, and anterior chamber paracentesis have been used with limited effectiveness and carry the possible risk of delaying appropriate management.^5^ Notably, a meta-analysis reported that anterior chamber paracentesis and ocular massage resulted in reduced visual acuity and recovery rates compared with no treatment and that administration of thrombolytic agents when administered >4.5 hours after onset was not effective.^6^ Recently, novel treatments such as thrombolytic agents and hyperbaric oxygen therapy (HBOT) are being investigated for the treatment of CRAO.^7^, ^8^, ^9^, ^10^ Although several clinical trials have reported on blood clot removal and neuroprotection,^11^, ^12^, ^13^ no treatment has yet been established to improve visual outcomes in patients with CRAO. Thus, there is an urgent need for novel drugs with improved efficacy to treat CRAO because this condition is acute and causes irreversible and severe visual dysfunction.

Calpains are calcium-activated cysteine proteases, crucial for maintaining cellular homeostasis through the controlled breakdown of substrate proteins. Evidence suggests the involvement of calpain activity in the pathology of several human diseases, including traumatic brain injuries, diabetes, muscular dystrophy, neurodegenerative diseases, cardiovascular diseases, and cancer. The effectiveness of calpain inhibitors in multiple diseases has been evaluated in preclinical studies.^14^, ^15^, ^16^ However, to the best of our knowledge, none of the calpain inhibitors have been approved as therapeutic agents to date.^14^, ^15^, ^16^ The underlying physiological mechanism of calpain in CRAO remains unclear.^17^ When the intracellular calcium concentration in retinal ganglion cells is abnormally increased by a severe stimulus, such as ischemia, it leads to excessive calpain activation, resulting in cell death.^18^ The death of retinal ganglion cells results in visual field narrowing and blindness. Furthermore, calpains are also involved in reperfusion injury leading to neuronal death.^18^

The investigational drug, SJP-0008, is a calpain inhibitor exhibiting selective and reversible action against calpain enzyme activity.^19^ Previous preclinical research has shown that calpains play a role in retinal ganglion cell death in a rat model of high intraocular pressure as well as in a hypoxic organ culture of monkey retinas, indicating a potential role for calpain inhibitors in treating CRAO.^20^^,^^21^ When taken orally, SJP-0008 (at 10 mg/kg) reaches a peak concentration of 300 nM in the retinal tissue within 1 hour in rats, decreasing to 100 nM within 8 hours.^22^ Oral administration of SJP-0008 was able to penetrate the retina and inhibit calpain-1 and calpain-2.^23^ Consequently, SJP-0008 showed a strong protective effect against axonal degeneration–induced retinal ganglion cell death, indicating that it is a promising therapeutic option for treating disorders leading to vision loss.^23^

A phase I study confirming that SJP-0008 is well-tolerated at a 300-mg dose administered orally for up to 1 week has already been completed (trial registration number: UMIN000019104; unpublished data). Investigating the efficacy and safety of SJP-0008 vs. a control is the required next step in the drug development process. However, the rapid, irreversible, and infrequent onset of visual field loss in CRAO makes it challenging to prospectively follow patients with CRAO as controls. This is an important practical challenge in designing clinical trials aimed at assessing the safety and efficacy of CRAO treatment; therefore, it is clinically significant to be able to utilize patients from a disease registry with high-quality data that can withstand regulatory filing as a control group for clinical trials. Thus, we prospectively developed a CRAO disease registry and used patients from the registry as a control group to compare the efficacy and safety of orally administered SJP-0008 in patients with CRAO in the Japanese population.

## Methods

### Study Design and Patients

This 2-part, single-center study was conducted between January 8, 2020, and July 16, 2021 (part 1), and July 10, 2020, and July 7, 2022 (part 2), at Tohoku University Hospital in Sendai, Japan. Part 1 was a physician-/investigator-initiated, phase IIa, randomized, double-blinded, parallel-group study that included patients diagnosed with CRAO who were randomized 1:1 using masked randomization to receive SJP-0008 at a dose of either 100 mg or 200 mg. Part 2 (CRAO registry; prospective cohort study) included patients diagnosed with CRAO who were not treated with SJP-0008 (non-SJP group).

Patients included in the study were ≥20 years of age, were diagnosed with nonarteritic CRAO, had provided written consent, were able to start participation in the clinical trial or the CRAO registry between 3 and 48 hours after the onset of CRAO, and had visual acuity in the target eye greater than or equal to hand motion and less than 0.1 (decimal visual acuity) in a screening examination before enrollment. The time window between 3 and 48 hours after the onset of CRAO was chosen to exclude transient CRAO (for <3 hours) because early reperfusion may prevent irreversible visual dysfunction and to include patients actively treated within 48 hours as recommended. In addition, patients with no prespecified hematological and blood biochemical abnormalities in pre-enrollment screening tests were included in part 1 of the study. Patients with retinal diseases in the target eye other than CRAO, such as diabetic retinopathy, retinal detachment, macular disease, or retinitis pigmentosa that could progress during the study period, as determined by the investigator or subinvestigator, were excluded from the study. However, patients were not excluded if the investigator or subinvestigator determined that the condition was unlikely to affect the efficacy and safety of the study drug. Patients with a history of internal ocular surgery (including laser therapy) in the target eye within 90 days before enrollment, patients with poorly controlled comorbidities, patients judged by the investigator or subinvestigator to be ineligible for participation, and patients who had participated in another clinical trial within 4 weeks before obtaining consent were not included in the study. In addition, in part 1 of the study, patients with visual acuity of <0.1 (decimal visual acuity) in the non-CRAO eye, patients unwilling to use an effective contraception method until 90 days (male patients) or 4 weeks (female patients) after the last dose, pregnant or nursing women, and patients who had previously received this investigational drug were excluded from the study. CYP3A4-modulating drugs were prohibited during the study period. In part 2, patients receiving unapproved or off-label treatments because of participation in clinical trials (including company clinical trials, investigator-initiated clinical trials, or expanded clinical trials), advanced medical care, or specific clinical research were excluded.

### Procedures

Patients were classified into 3 groups: 2 groups received SJP-0008 (at doses of 100 mg or 200 mg) and 1 group did not receive SJP-0008 (non-SJP group, from the nonrandomized registry cohort). Treatment allocation was performed using a central registration method via an electronic data capture system. The allocation table was implemented in the system according to predecided work procedures prepared by the person responsible for allocating the investigational drug. The study drugs were prepared to be indistinguishable from one another. The trial was blinded to all individuals involved (including patients and the investigator), except for the study drug assignment manager. Patients in the 100-mg SJP-0008 group received 1 × 100-mg tablet of SJP-0008 and 1 × placebo tablet once daily for 4 weeks (maximum 29 days); patients in the 200-mg SJP-0008 group received 2 × 100-mg tablets of SJP-0008 once daily for 4 weeks (maximum 29 days). SJP-0008 treatment was initiated at least 3 hours but no more than 48 hours after the onset of CRAO. The 12-week study duration included a 4-week drug administration period (up to 29 days following initiation), followed by an 8-week postobservation period. The non-SJP group was observed prospectively for 12 weeks without receiving SJP-0008 or placebo tablets; standard treatments were not prohibited.

### Assessments and Endpoints

The primary endpoint of the study was to assess changes in ETDRS visual acuity at 12 weeks in the target eye of patients with CRAO after SJP-0008 treatment. Secondary endpoints included evaluation of (1) change in ETDRS visual acuity, (2) change in retinal sensitivity and central visual field, (3) change in perfusion status of the retinal artery, (4) change in blood flow in the occluded retinal artery, and (5) change in foveal thickness. A refraction test was performed as needed before visual acuity testing. Visual field testing was performed to assess the foveal threshold, the average values of the central 4 points, the average values of the central 12 points, and mean deviation (MD) using the 10-2 program of the Humphrey Visual Field Analyzer (Carl Zeiss Meditec). Mean deviation represents the mean difference from the age-adjusted expected value. Retinal sensitivity was measured using microperimetry (MP-3; Nidek) for the average value of the central 4 points, the average value of the central 12 points, and the average of all points. MP-3 measurements were carried out using the 4-2 full-threshold staircase strategy with the standard Goldmann III stimulus size and 68 test points positioned identically to those in the Humphrey visual field analyzer 10-2 test grid. The perfusion status of the retinal artery was assessed using fluorescence angiography. OCT (Cirrus 5000 HD-OCT, Carl Zeiss Meditec; macular cube protocol) was used to evaluate the foveal thickness of the retina. Visual acuity, the visual field, and OCT were measured at screening and at 1, 2, 4, 8, and 12 weeks following initiation of the study drug as well as at trial discontinuation. Microperimetry with MP-3 was performed at screening and at 1, 2, 4, and 12 weeks after initiation of the study drug as well as at trial discontinuation. Fluorescence angiography was performed at screening and at 1 and 4 weeks. Fluorescence angiography was also conducted on day 2 for patients who were able to undergo the test.

The safety of SJP-0008 oral treatment was assessed by recording the incidence and severity of adverse events (AEs). All unfavorable events related to the drug during the 12-week observation period of the study were recorded.

### Statistical Analysis

The sample size for part 1 of the trial was set at 20 patients, informed by the known annual incidence of CRAO of 1.8 to 1.9 per 100 000 persons^24^^,^^25^ and the feasibility of the trial.

The statistical analysis was conducted according to a predecided statistical analysis plan after data collection. For the primary efficacy endpoint (i.e., change in ETDRS visual acuity in the target eye), a general linear model was fit using the difference in ETDRS visual acuity at screening and at each observation as the outcome variable, allowing for repeated measurements of the same patient, including group, time point, visual acuity at screening, and group × time point interaction terms. Robust variance was used, and the working correlation matrix was analyzed using the compound symmetry structure for comparisons between groups. Secondary efficacy endpoints were compared between groups using a general linear model in the efficacy analysis population. Quantitative variables were summarized using mean and standard deviation (SD) and mean and standard error (SE), and categorical variables were summarized using frequency and percentage. All statistical tests used a 2-sided significance level of 0.05 and were performed using the SAS software (version 9.4; SAS Institute Inc.).

This study (part 1: jRCT2021190013 and part 2: jRCT1020190019) was conducted in accordance with the Declaration of Helsinki,^26^ the Ministerial Ordinance on Standards for the Conduct of Clinical Trials on Pharmaceuticals, and the Ethical Guidelines for Medical and Health Research Involving Human Subjects. Written informed consent forms were obtained from all patients before their participation in the clinical trial. The study protocol was approved by the Institutional Review Board of Tohoku University Hospital and the Ethics Committee of the Graduate School of Medicine, Tohoku University.

## Results

**Figure 1:**
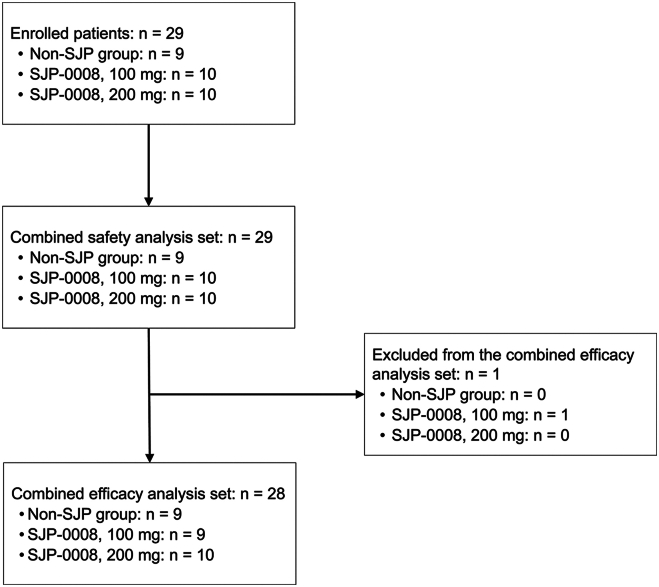
Patient inclusion flowchart.

In the 100-mg and 200-mg SJP-0008 groups, 25 patients were screened, and 20 were randomized. In the non-SJP group, 10 patients were screened, and 9 were included in the analysis. Figure 1 shows a flowchart of patient inclusion. The safety analysis group included 10 patients who received 100 mg of SJP-0008 and 10 patients who received 200 mg of SJP-0008. The efficacy analysis group included 9 patients who received 100 mg of SJP-0008, 10 patients who received 200 mg of SJP-0008, and 9 patients who did not receive SJP.

The baseline characteristics of the included patients are summarized in Table 1. The mean ± SD age of the patients was 68.8 ± 14.9 years, and male patients comprised 78.6% of the study population. Mean ± SD ETDRS visual acuity at baseline was 4.9 ± 8.3 letters, and the mean ± SD foveal threshold was 4.8 ± 8.4 dB. In the previous 2 years, 7 (25.0%) patients had diabetes, 23 (82.2%) patients had hypertension, and 14 (50%) patients had dyslipidemia. The patients were administered antiplatelets, anticoagulation therapy, and circulation-improving therapy, such as kallidinogenase and ocular massage before the study and during the study period. The patient-wise details of treatments are summarized in Table S1 (available at www.ophthalmologyscience.org).

**Table 1:** Baseline Characteristics of Included Patients

Group	SJP-0008, 100 mg (n = 9)	SJP-0008, 200 mg (n = 10)	Non-SJP (n = 9)	Overall (n = 28)
Mean age (yrs) ± SD	69.80 ± 19.61	67.40 ± 13.58	69.20 ± 12.40	68.80 ± 14.90
Male, n (%)	7 (77.8)	7 (70.0)	8 (88.9)	22 (78.6)
Duration after CRAO onset, n (%)				
≤12 hrs	2 (22.2)	4 (40.0)	3 (33.3)	9 (32.1)
12–24 hrs	4 (44.4)	3 (30.0)	3 (33.3)	10 (35.7)
>24 hrs	3 (33.3)	3 (30.0)	3 (33.3)	9 (32.1)
Mean ETDRS (letters) ± SD	4.8 ± 7.3	6.4 ± 8.4	3.4 ± 9.6	4.9 ± 8.3
Mean logMAR (value) ± SD	2.44 ± 0.56	1.91 ± 0.74	2.44 ± 0.59	2.25 ± 0.67
Mean foveal threshold (Humphrey value) ± SD	4.3 ± 7.2	8.4 ± 11.2	1.3 ± 4.0	4.8 ± 8.4
Presence of cilioretinal artery, n (%)	2 (22.2)	0 (0.0)	2 (22.2)	4 (14.3)
Presence of reperfusion, n (%)	6 (66.7)	6 (60.0)	5 (55.6)	17 (60.7)
Presence of diabetes, n (%)	1 (11.1)	3 (30.0)	3 (33.3)	7 (25.0)
Presence of hypertension, n (%)	8 (88.9)	7 (70.0)	8 (88.9)	23 (82.2)
Presence of dyslipidemia, n (%)	3 (33.3)	5 (50.0)	6 (66.7)	14 (50.0)

CRAO = central retinal artery occlusion; logMAR = logarithm of the minimum angle of resolution; SD = standard deviation.

### Efficacy Analysis

**Figure 2:**
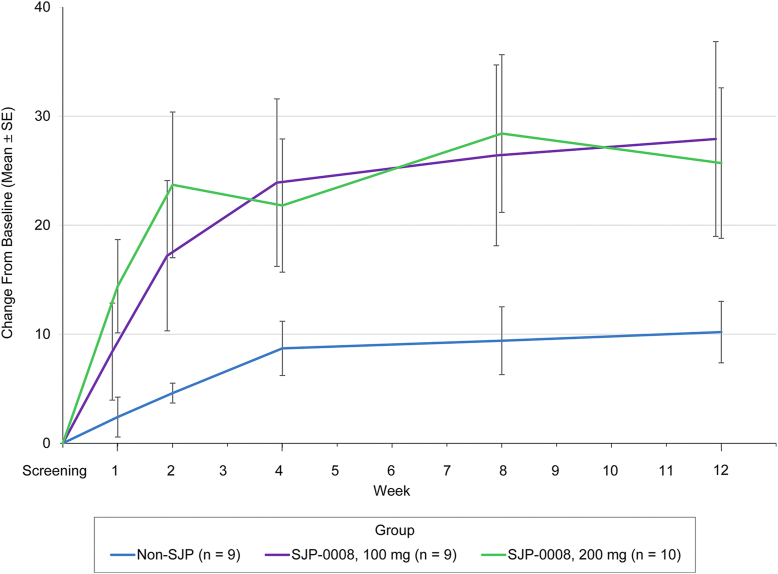
Mean change versus baseline in ETDRS visual acuity in the SJP-0008 groups at different time points. Change in ETDRS visual acuity scores from baseline (screening) to week 12 in both the SJP-0008 treatment groups and the non-SJP control group. Data are expressed as the mean change in letters read ± SE. SE = standard error.

The primary efficacy endpoint, change in ETDRS visual acuity in the target eye from baseline to week 12, was markedly higher in the 100-mg SJP-0008 group (27.9 letters) and 200-mg group (25.7 letters) than in the non-SJP group (10.2 letters) (Table 2, Fig 2, and Fig S1, available at www.ophthalmologyscience.org). The difference (mean ± SE) in improvement at 12 weeks from baseline was significantly greater in the 200-mg SJP-0008 group than in the non-SJP group (15.5 ± 7.45 letters; *P* = 0.040). At week 12, in the combined SJP-0008 treatment group (n = 19), 5 patients (26.3%) achieved a final visual acuity >20/100, and 9 patients (47.4%) achieved >20/200.

**Table 2:** Change in ETDRS Visual Acuity in the SJP-0008 Groups vs. the Non-SJP Group at Different Time Points

Visit	ETDRS Letters			Mean Change from Screening (ETDRS Letters)		
	SJP-0008, 100 mg (n = 9)	SJP-0008, 200 mg (n = 10)	Non-SJP (n = 9)	SJP-0008, 100 mg (n = 9)	SJP-0008, 200 mg (n = 10)	Non-SJP (n = 9)
Screening	4.80 ± 2.29 (0.25 to 9.31)	6.40 ± 2.53 (1.39 to 11.41)	3.40 ± 3.02 (–2.53 to 9.42)	-	-	-
Wk 1	13.00 ± 4.34 (4.41 to 21.59)	21.80 ± 6.03 (9.85 to 33.75)	4.70 ± 3.37 (–2.02 to 11.35)	8.40 ± 4.45 (–0.42 to 17.26)	14.40 ± 4.28 (5.90 to 22.92)	2.40 ± 1.83 (–1.24 to 6.03)
Wk 2	21.80 ± 6.34 (9.23 to 34.33)	31.10 ± 8.36 (14.54 to 47.66)	7.10 ± 5.16 (–3.07 to 17.37)	17.20 ± 6.89 (3.52 to 30.87)	23.70 ± 6.68 (10.44 to 36.97)	4.60 ± 0.91 (2.83 to 6.43)
Wk 4	28.40 ± 8.50 (11.61 to 45.28)	29.20 ± 7.68 (13.99 to 44.41)	11.00 ± 7.37 (–3.59 to 25.59)	23.90 ± 7.68 (8.61 to 39.11)	21.80 ± 6.11 (9.68 to 33.94)	8.70 ± 2.49 (3.79 to 13.67)
Wk 8	31.00 ± 8.78 (13.61 to 48.39)	35.80 ± 8.54 (18.89 to 52.71)	12.50 ± 8.63 (–4.63 to 29.57)	26.40 ± 8.29 (9.95 to 42.89)	28.40 ± 7.23 (14.05 to 42.76)	9.40 ± 3.11 (3.21 to 15.56)
Wk 12	32.60 ± 9.26 (14.23 to 50.92)	33.10 ± 8.58 (16.10 to 50.10)	12.40 ± 7.61 (–2.63 to 27.51)	27.90 ± 8.93 (10.14 to 45.61)	25.70 ± 6.90 (12.02 to 39.40)	10.20 ± 2.82 (4.58 to 15.76)
Difference versus the non-SJP group at 12 wks	20.10 ± 11.99 (–3.61 to 43.87) *P* = 0.096	20.70 ± 11.47 (–2.06 to 43.37) *P* = 0.074		17.70 ± 9.55 (–1.26 to 36.66) *P* = 0.067	15.50 ± 7.45 (0.74 to 30.34) *P* = 0.040	

CI = confidence interval; SE = standard error.

Data are expressed as letters ± SE (95% CI).

All estimates and *P* values were calculated using a general linear model with a compound symmetry working correlation matrix and robust variance estimators.

The secondary efficacy endpoint, Humphrey visual field test results (i.e., foveal threshold) in the target eye, showed a difference of 7.2 ± 4.20 (*P* = 0.091) between the 100-mg SJP-0008 group and the non-SJP group and a statistically significant difference of 12.2 ± 3.93 (*P* = 0.002) between the 200-mg SJP-0008 group and the non-SJP group at 12 weeks (Table 3). Although MD in the target eye in the non-SJP group remained practically unchanged from baseline, both SJP-0008 groups demonstrated significant improvement in MD at 12 weeks (Table 3). At 12 weeks, the 100-mg and 200-mg SJP-0008 groups showed a statistically significant difference in MD compared with the non-SJP group, with differences of 8.74 ± 4.12 and 8.12 ± 4.03, respectively (*P* = 0.036, *P* = 0.046; Table 3). For the central 12-point mean and central 4-point mean, the 200-mg SJP-0008 group showed statistically significant differences of 7.55 ± 3.06 (*P* = 0.015) and 7.00 ± 2.64 (*P* = 0.010), respectively, after 12 weeks compared with the non-SJP group (Table 4). Similarly, the 100-mg SJP-0008 group exhibited differences of 7.48 ± 3.71 (*P* = 0.046) and 6.39 ± 3.51 (*P* = 0.072) for the central 12-point mean and central 4-point mean, respectively, compared with the non-SJP group (Table 4).

**Table 3:** Humphrey Visual Field Test (Foveal Threshold and MD) in the SJP-0008 Groups vs. the Non-SJP Groups at Different Time Points

Visit	Foveal Threshold			MD		
	SJP-0008, 100 mg (n = 9)	SJP-0008, 200 mg (n = 10)	Non-SJP (n = 9)	SJP-0008, 100 mg (n = 9)	SJP-0008, 200 mg (n = 10)	Non-SJP (n = 9)
Screening	4.3 ± 2.25 (–0.12 to 8.79)	8.4 ± 3.35 (1.76 to 15.04)	1.3 ± 1.26 (–1.16 to 3.82)	–30.69 ± 1.81 (–34.27 to –27.10)	–24.95 ± 3.22 (–31.32 to –18.57)	–31.97 ± 1.22 (–34.39 to –29.55)
Wk 1	8.1 ± 2.68 (2.81 to 13.41)	4.8 ± 2.34 (0.16 to 9.44)	0.7 ± 0.63 (–0.58 to 1.91)	–27.40 ± 2.26 (–31.89 to –22.92)	–26.88 ± 2.88 (–32.59 to –21.17)	–32.85 ± 0.73 (–34.29 to –31.40)
Wk 2	11.0 ± 3.45 (4.17 to 17.83)	11.7 ± 3.84 (4.09 to 19.31)	3.5 ± 2.07 (–0.54 to 7.64)	–24.61 ± 2.51 (–29.57 to –19.64)	–25.57 ± 2.97 (–31.46 to –19.69)	–31.58 ± 1.56 (–34.67 to –28.48)
Wk 4	12.4 ± 3.29 (5.93 to 18.96)	12.6 ± 3.77 (5.14 to 20.06)	4.3 ± 2.15 (0.07 to 8.60)	–23.79 ± 2.81 (–29.37 to –18.22)	–24.14 ± 3.23 (–30.53 to –17.76)	–31.62 ± 1.19 (–33.97 to –29.26)
Wk 8	11.9 ± 3.83 (4.31 to 19.47)	11.0 ± 3.98 (3.12 to 18.88)	3.4 ± 2.12 (–0.80 to 7.61)	–22.93 ± 3.40 (–29.67 to –16.19)	–24.26 ± 3.33 (–30.85 to –17.67)	–31.56 ± 1.47 (–34.47 to –28.66)
Wk 12	8.4 ± 4.04 (0.39 to 16.39)	13.4 ± 3.76 (5.96 to 20.84)	1.2 ± 1.15 (–1.06 to 3.50)	–21.79 ± 3.53 (–28.80 to –14.79)	–22.41 ± 3.43 (–29.21 to –15.61)	–30.53 ± 2.11 (–34.70 to –26.36)
Difference versus the non-SJP group at 12 wks	7.2 ± 4.20 (–1.15 to 15.48), *P* = 0.091	12.2 ± 3.93 (4.40 to 19.96), *P* = 0.002		8.74 ± 4.12 (0.59 to 16.89), *P* = 0.036	8.12 ± 4.03 (0.15 to 16.10), *P* = 0.046	

CI = confidence interval; MD = mean deviation; SE = standard error.

Data are expressed as estimates ± SE (95% CI).

All estimates and *P* values were calculated using a general linear model with a compound symmetry working correlation matrix and robust variance estimators.

**Table 4:** Secondary Endpoints

Additional Secondary Outcome	Screening			Week 12			Difference from the Non-SJP Group at 12 Weeks	
	SJP-0008, 100 mg (n = 9)	SJP-0008, 200 mg (n = 10)	Non-SJP (n = 9)	SJP-0008, 100 mg (n = 9)	SJP-0008, 200 mg (n = 10)	Non-SJP (n = 9)	SJP-0008, 100 mg	SJP-0008, 200 mg
Visual field test∗								
Central 12 points mean value	2.05 ± 1.33 (–0.60 to 4.69)	7.65 ± 3.09 (1.54 to 13.76)	1.06 ± 0.89 (–0.71 to 2.82)	9.45 ± 3.32 (2.87 to 16.03)	9.52 ± 2.58 (4.42 to 14.63)	1.97 ± 1.65 (–1.29 to 5.23)	7.48 ± 3.71 (0.14 to 14.82) *P* = 0.046	7.55 ± 3.06 (1.50, 13.61) *P*= 0.015
Central 4 points mean value	1.89 ± 1.24 (–0.58 to 4.35)	6.95 ± 3.21 (0.59 to 13.31)	0.67 ± 0.60 (–0.52 to 1.85)	8.06 ± 3.20 (1.72 to 14.39)	8.63 ± 2.21 (4.25 to 13.00)	1.67 ± 1.46 (–1.22 to 4.55)	6.39 ± 3.51 (–0.57 to 13.35) *P* = 0.072	7.00 ± 2.64 (1.72 to 12.20) *P* = 0.010
Retinal sensitivity test∗								
Central 4 points mean value	0.67 ± 0.44 (–0.19 to 1.54)	3.78 ± 2.30 (–0.80 to 8.35)	0.0	4.71 ± 2.65 (–0.56 to 9.98)	3.48 ± 1.59 (0.32 to 6.63)	0.17 ± 0.16 (–0.15 to 0.48)	4.54 ± 2.66 (–0.74 to 9.82) *P* = 0.091	3.31 ± 1.60 (0.13 to 6.48) *P* = 0.041
Central 12 points mean value	0.91 ± 0.64 (–0.36 to 2.19)	4.49 ± 2.56 (–0.60 to 9.57)	0.0	5.32 ± 2.39 (0.57 to 10.06)	4.62 ± 1.82 (1.00 to 8.24)	0.69 ± 0.63 (–0.56 to 1.93)	4.63 ± 2.47 (–0.28 to 9.54) *P* = 0.064	3.93 ± 1.93 (0.10 to 7.76) *P* = 0.044
All test points mean value	1.75 ± 1.12 (–0.48 to 3.97)	5.60 ± 2.88 (–0.13 to 11.32)	0.45 ± 0.34 (–0.23 to 1.14)	7.72 ± 2.46 (2.84 to 12.61)	6.33 ± 2.46 (1.44 to 11.22)	1.86 ± 1.27 (–0.67 to 4.39)	5.86 ± 2.77 (0.36 to 11.36) *P* = 0.037	4.47 ± 2.77 (–1.04 to 9.97) *P* = 0.111
Foveal retinal thickness∗ (μm)	319.80 ± 37.52 (245.46 to 394.10)	381.70 ± 43.13 (296.27 to 467.13)	398.1 ± 25.62 (347.37 to 448.85)	225.8 ± 6.24 (213.40 to 238.11)	234.10 ± 9.92 (214.46 to 253.74)	249.10 ± 5.12 (238.95 to 259.25)	–23.3 ± 8.07 (–39.33 to –7.35) *P* = 0.005	–15.0 ± 11.16 (–37.10 to 7.11) *P* = 0.182

	Screening			Week 4			Difference from the Non-SJP Group at 4 Weeks	
	SJP-0008, 100 mg (n = 9)	SJP-0008, 200 mg (n = 10)	Non-SJP (n = 9)	SJP-0008, 100 mg (n = 9)	SJP-0008, 200 mg (n = 10)	Non-SJP (n = 9)	SJP-0008, 100 mg	SJP-0008, 200 mg
Fluorescein angiography†								
Patients with reperfusion	66.70% (35.87% to 97.46%)	60.0% (29.64% to 90.36%)	55.60% (23.09% to 88.02%)	88.90% (68.36% to 100.0%)	80.0% (55.21% to 100.0%)	77.80% (50.62% to 100.0%)	11.10% (–22.94% to 45.16%) *P* = 0.522	2.20% (–34.55% to 39.00%) *P* = 0.906
Arm-to-retina circulation time (sec)	45.38 ± 9.90 (25.52 to 65.25)	117.19 ± 66.57 (–16.33 to 250.72)	138.16 ± 50.08 (37.70 to 238.61)	30.07 ± 8.23 (13.56 to 46.57)	40.55 ± 12.43 (15.63 to 65.48)	31.51 ± 2.90 (25.70 to 37.33)	–1.44 ± 8.72 (–18.94 to 16.05) *P* = 0.869	9.04 ± 12.76 (–16.55 to 34.64) *P* = 0.482

CI = confidence interval; SE = standard error.

Data are expressed as estimates ± SE (95% CI).

∗ All estimates and *P* values were calculated using a general linear model with a compound symmetry working correlation matrix and robust variance estimators.

† All estimates and *P* values were calculated using a generalized linear model with a binomial distribution and an identity link function, along with a compound symmetry working correlation matrix and robust variance estimators.

**Figure 3:**
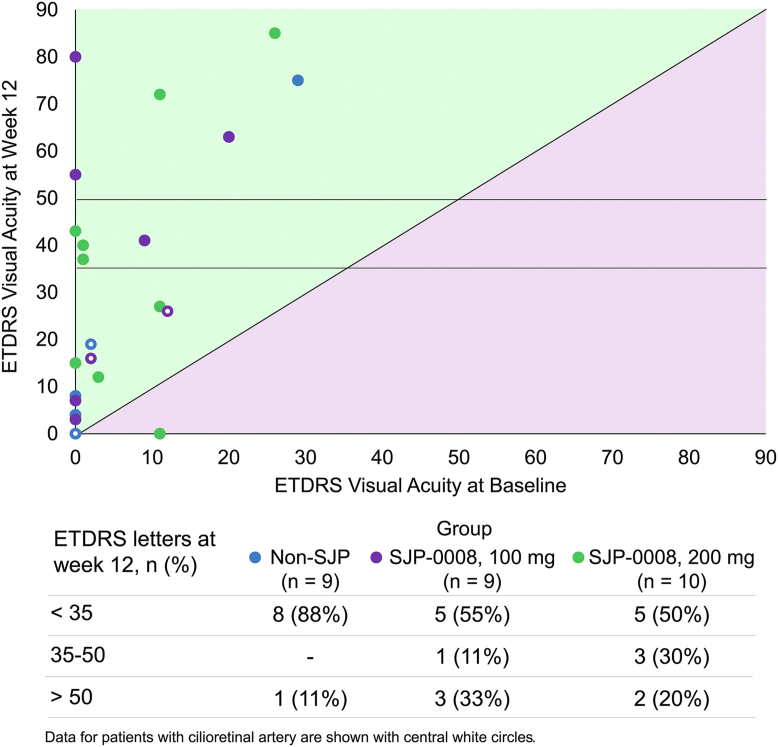
ETDRS visual acuity improvement in individual patients at week 12.

The results for the additional secondary endpoints are summarized in Table 4. The mean retinal sensitivity in the central 4 points was higher by 3.31 dB in the 200-mg SJP-0008 group than in the non-SJP group, and the difference was statistically significant (*P* = 0.041). Similarly, the mean retinal sensitivity in the central 12 points was significantly higher in the 200-mg SJP-0008 group than in the non-SJP group (*P* = 0.044). Furthermore, the mean sensitivity across all test points in the 100-mg SJP-0008 group was 5.86 dB higher than that in the non-SJP group, and the difference was statistically significant (*P* = 0.037). There were no significant differences between groups for the percentage of patients with reperfusion or arm-to-retina circulation time on fluorescein angiography. At week 12, foveal thickness (mean ± SE) as measured by OCT was 225.8 ± 6.24 μm, 234.10 ± 9.92 μm, and 249.10 ± 5.12 μm in the 100-mg SJP-0008 group, 200-mg SJP-0008 group, and non-SJP-0008 group, respectively. Notably, the difference in foveal thickness between the 100-mg SJP-0008 group and the non-SJP group was statistically significant (*P* = 0.005). Logarithm of the minimum angle of resolution (LogMAR) visual acuity in the target eye at baseline and week 12 is reported in Table S2 (available at www.ophthalmologyscience.org). ETDRS visual acuity improvement in individual patients at week 12 is reported in Figure 3.

### Safety Evaluation

Two events of cerebral infarction in patients who received 100 mg of SJP-0008 were considered serious AEs; however, these AEs were not considered to be related to the study drug and resolved with treatment (Table S3, available at www.ophthalmologyscience.org). One patient with cerebral infarction was deemed ineligible to continue the study. Ocular AEs included 1 case of allergic conjunctivitis that required treatment; other events resolved spontaneously and did not require treatment or discontinuation of the study drug. No causal relationship to the study drug was noted. The incidence of AEs related to ocular injury, such as papillary hemorrhage, retinal hemorrhage, vitreous hemorrhage, and papillary neovascularization, was similar between the 100-mg and 200-mg SJP-0008 groups. These ocular hemorrhagic events may be secondary to ischemia-driven neovascularization.^27^, ^28^, ^29^ None of these events resulted in study drug discontinuation. No clinical abnormalities related to laboratory values and vital signs were observed (Table S3).

## Discussion

SJP-0008, an oral calpain inhibitor, showed both safety and efficacy in this phase IIa study. A 4-week treatment improved ETDRS visual acuity in the target eyes of patients with CRAO, with the improvement sustained until 12 weeks. Humphrey visual field scores also showed notable improvement. Reported AEs, such as cerebral infarction and ocular damage, were not associated with the study drug, indicating a favorable overall safety profile.

Several randomized clinical trials have been conducted to evaluate treatments for CRAO; however, no drug has demonstrated adequate therapeutic efficacy. Recent findings from a large cohort of 145 CRAO eyes assessed with multimodal imaging reported visual improvement of ≥2 lines in 27% of eyes with cilioretinal artery (CLRA) sparing and 36% in non-CLRA eyes. However, final visual acuity ≥20/200 was achieved in only 17% of CLRA-sparing eyes and 4% of non-CLRA eyes.^30^ Thrombolytic therapy was evaluated in a prospective randomized clinical trial (the European Assessment Group for Lysis in the Eye study), in which local intra-arterial thrombolysis using a recombinant tissue plasminogen activator was compared with conservative standard treatment. However, the study was terminated after an interim analysis because the 2 treatment drugs had similar efficacy and local intra-arterial thrombolysis had safety concerns, with a higher rate of AEs.^13^ In another study, a phase I/II trial of an inhibitor of the valosin-containing protein called Kyoto University Substance 121, significant improvement in best-corrected visual acuity was observed in all 9 patients after they received intravitreal injections^11^; however, the results need to be interpreted with caution because of the lack of a comparison arm. In addition, whereas SJP-0008 is administered orally, Kyoto University Substance 121 requires intravitreal administration by a physician in a hospital. In another retrospective study, the outcomes of HBOT were assessed in 41 patients diagnosed with CRAO. There was a statistically significant improvement in visual acuity after HBOT compared with presentation (median [interquartile range] logMAR visual acuity: 1.94 [1.00 to 2.00] versus 2.00 [1.70 to 2.30]) in patients with CRAO. It was observed that, among patients who received early HBOT (i.e., within 9 hours; n = 20), 14 (70%) had clinically meaningful improvement with a final logMAR visual acuity ≥0.3.^10^

In a recently reported meta-analysis of CRAO therapies, the proportion of patients with logMAR visual acuity improved to ≤0.7 (from a baseline of ≥1.0) was 17.7% (95% confidence interval: 13.9%, 21.4%) among natural history studies versus 7.4% (95% confidence interval: 3.7%, 11.1%) among studies of conservative treatments that included ocular massage, anterior chamber paracentesis, and hemodilution.^6^ In the European Assessment Group for Lysis in the Eye study, baseline logMAR visual acuity in patients who received conservative treatment or fibrinolytic therapy was 2.11 and 2.18, respectively, with logMAR visual acuity at 1 month of 1.67 and 1.74, respectively^13^; the proportion of patients with logMAR visual acuity of ≥1.0 at 1 month was 15% and 16.7%, respectively.^13^ In the combined analyses of the present study, the baseline logMAR visual acuities for the 100-mg SJP-0008 group, the 200-mg SJP-0008 group, and the non-SJP group were 2.44, 1.91, and 2.44, respectively, and the 4-week logMAR visual acuities were 1.23, 1.10, and 1.91, respectively (Table S2). In addition, the proportion of patients with decimal visual acuity ≥0.1, equivalent to logMAR visual acuity of 1.0, at 4 weeks was 33.3% in the 100-mg SJP-0008 group, 50.0% in the 200-mg SJP-0008 group, and 11.1% in the non-SJP group. This visual improvement in the non-SJP group in this study is comparable to that previously reported for conservative treatment, whereas the SJP-treated groups showed marked improvement in visual acuity. Thus, it is reasonable to suggest that SJP-0008 demonstrated greater improvement than the nonrandomized registry cohort, the non-SJP group. These findings should be validated in larger-scale, multicenter randomized controlled trials.

Overall, SJP-0008 was well-tolerated. Two cases of cerebral infarction were reported as serious AEs. However, a causal relationship between these events and the investigational drug was ruled out, and symptoms improved with treatment. Furthermore, no dose–response effect was observed. In a previous report comprising approximately 17 000 patients with CRAO, the incidence of in-hospital stroke was reported to be 12.9%.^1^ The occurrence of cerebral infarction is consistent with previous studies of patients with CRAO. With the exception of 1 case of allergic conjunctivitis without a proven causal relationship to the study drug, no other AEs requiring discontinuation of the study drug or medical intervention were observed, and there were no other clinically significant AEs.

Calpain inhibitors exert a neuroprotective effect on retinal ganglion cells by suppressing cell death.^20^ In this clinical trial, administration of SJP-0008 in patients with CRAO helped prevent visual loss. This suggests that in CRAO, an ischemic disorder, SJP-0008 may have inhibited calpain activation because of calcium influx in retinal ganglion cells, thereby inhibiting cell death through this mechanism.

A previous study of rhesus monkeys reported irreversible morphological and functional retinal damage when ischemia lasted longer than 4 hours.^31^ Although the European Assessment Group for Lysis in the Eye study, which used thrombolytic agents, was conducted on patients within 20 hours of ischemia onset, the study could not confirm the efficacy of the treatment.^13^ A subsequent prospective case series suggested that thrombolysis may need to be initiated within a 4.5-hour therapeutic window from CRAO onset in order to be effective.^32^ In contrast, in the present clinical trial of SJP-0008, visual acuity improved even in patients who initiated treatment more than 24 hours after CRAO onset (data not shown). It has been reported that in most patients with CRAO, the retinal circulatory status improves to some degree within a few days.^11^ In this study, approximately 80% of patients showed reperfusion by the end of the SJP-0008 treatment period. This suggests that if there are surviving retinal ganglion cells within 48 hours of CRAO onset, SJP-0008 may help preserve visual function by inhibiting calpain activation associated with reperfusion.

The main limitation of this study is the relatively small sample size, which may restrict the generalizability of the findings and limit the statistical power to detect subtle treatment effects. We have not used formal sample size estimation but instead relied on feasibility. However, considering that CRAO is a rare disease, a sample size of 29 may be considered sufficient for a phase IIa exploratory study to determine preliminary efficacy and safety of SJP-0008 in patients with CRAO. A subsequent phase III randomized controlled trial (jRCT2011230042; ongoing) was designed to validate the results in a larger patient population.^33^ Furthermore, the selection of the control group was one of the critical aspects of the study. However, considering the acute nature and rarity of the disease, it was not feasible to select patients randomly from the registry for the control group of the present phase IIa trial. Specifically, CRAO is an ophthalmic emergency with a narrow therapeutic window, and patients typically have variable timing following symptom onset. Hence, the patients who presented to the clinic after symptom onset were assessed, and only those who met the eligibility criteria were registered in the CRAO registry. Selection bias is likely present in rare diseases, which may affect the generalizability of the results. However, this was a phase II clinical trial with safety as the primary endpoint. Furthermore, multiple patients showed improvement compared with previous reports. Thus, despite the selection bias, these findings indicate the value of proceeding to a large, multicenter trial in the future. Additionally, patients received treatments such as antiplatelets, anticoagulation therapy, and circulation-improving therapies (e.g., kallidinogenase) as well as ocular massage before and during the study period. The contribution of these treatments to the improvement of vision cannot be entirely ruled out. Overall, the nonrandomized and non–placebo-controlled nature of this study should be considered a major limitation. Future trials are needed to gather more data and identify additional safety concerns, if any. It is important to acknowledge that this study does not establish a relationship between structural changes in the retina and visual function. Future long-term follow-up studies, particularly those analyzing the ganglion cell layer, may provide valuable insights.

In conclusion, the results of the present first-in-patient study provide evidence of the safety and efficacy of SJP-0008 for the treatment of CRAO. Moreover, the SJP-0008 groups showed greater improvement compared with a prospective, nonrandomized registry cohort, the non-SJP group. These exploratory findings, although promising, are hypothesis-generating and require definitive confirmation in the ongoing randomized controlled trial.

## Data Availability

The data that support the findings of this study are available from the corresponding author, T.N., upon reasonable request.
