# Supplementary material for: Safety and Efficacy of Orally Administered SJP-0008 in Central Retinal Artery Occlusion: A Phase IIa Randomized Clinical Trial
Source: Ophthalmol Sci. 2025 Oct 10;6(1):100965. doi: 10.1016/j.xops.2025.100965 (PMC12662090; doi:10.1016/j.xops.2025.100965)

Supplementary Figure S1. Mean ETDRS visual acuity in the SJP-0008 groups and non-SJP control group at different time points.

Mean ETDRS visual acuity scores up to week 12 in both the SJP 0008 treatment groups and the non-SJP control group. Data are expressed as the mean ± SE

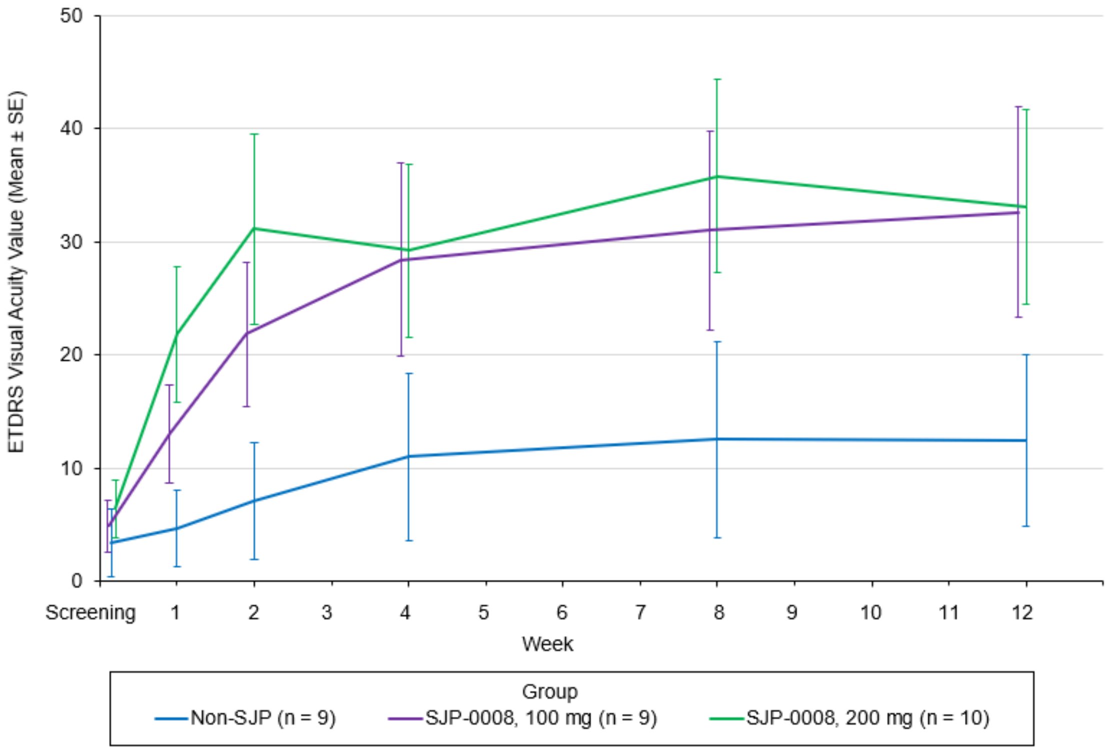

Supplement: Figure S1 [file mmc1.pdf]
