# Supplementary material for: Safety and Efficacy of Orally Administered SJP-0008 in Central Retinal Artery Occlusion: A Phase IIa Randomized Clinical Trial
Source: Ophthalmol Sci. 2025 Oct 10;6(1):100965. doi: 10.1016/j.xops.2025.100965 (PMC12662090; doi:10.1016/j.xops.2025.100965)
Supplement: Table S1 [file mmc2.pdf]

Supplementary Table S1. Standard Treatment Administered to Patients Before Enrolment.

Group	Clinical trial ID	Antiplatelet agents		Anticoagulants		Circulation-improving drugs		Ocular massage	
		Before enrollment	12-week study period	Before enrollment	12-week study period	Before enrollment	12-week study period	Before enrollment	12-week study period
SJP-0008, 100 mg (n = 9)	SJP100-1	Yes	Yes	No	No	No	No	Yes	No
	SJP100-2	No	Yes	No	Yes	No	No	No	No
	SJP100-3	No	No	No	No	No	No	Yes	No
	SJP100-4	No	No	No	No	No	No	No	No
	SJP100-5	No	No	No	No	No	No	Yes	No
	SJP100-6	No	No	No	No	No	No	No	No
	SJP100-7	No	No	No	No	No	No	Yes	No
	SJP100-8	Yes	Yes	No	No	No	No	Yes	No
	SJP100-9	No	No	No	No	No	No	No	No

**SJP-0008, 200 mg (n = 10)**	SJP200-1	Yes	Yes	No	No	No	No	No	No
	SJP200-2	No	No	No	No	No	No	No	No
	SJP200-3	No	No	No	No	No	No	Yes	No
	SJP200-4	No	No	No	No	No	No	No	No
	SJP200-5	No	No	No	No	No	No	Yes	No
	SJP200-6	No	No	No	No	No	No	No	No
	SJP200-7	No	No	No	No	No	No	Yes	No
	SJP200-8	No	No	No	No	No	No	Yes	No
	SJP200-9	No	No	No	No	No	No	Yes	No
	SJP200-10	No	No	No	No	No	No	Yes	No
**Non-SJP (n = 9)**	Registry-1	No	No	Yes	Yes	No	No	Yes	No
	Registry-2	Yes	Yes	No	No	No	No	Yes	No

	Registry-3	No	No	No	No	No	No	Yes	No
	Registry-4	Yes	Yes	No	No	No	No	Yes	No
	Registry-5	No	No	No	No	No	No	Yes	No
	Registry-6	Yes	Yes	No	No	No	No	Yes	No
	Registry-7	Yes	Yes	No	No	No	No	Yes	No
	Registry-8	No	No	No	No	No	No	No	No
	Registry-9	No	No	No	No	No	Yes	Yes	No
