# Supplementary material for: Safety and Efficacy of Orally Administered SJP-0008 in Central Retinal Artery Occlusion: A Phase IIa Randomized Clinical Trial
Source: Ophthalmol Sci. 2025 Oct 10;6(1):100965. doi: 10.1016/j.xops.2025.100965 (PMC12662090; doi:10.1016/j.xops.2025.100965)
Supplement: Table S2 [file mmc3.pdf]

Supplementary Table S2. LogMAR Visual Acuity in the Target Eye.

Visit	LogMAR visual acuity		
	**SJP-0008, 100 mg (n = 9)**	**SJP-0008, 200 mg (n = 10)**	**Non-SJP (n = 9)**
Screening	2.44 ± 0.18 (2.10, 2.79)	1.91 ± 0.22 (1.47, 2.35)	2.44 ± 0.19 (2.08, 2.81)
Week 1	1.74 ± 0.26 (1.23, 2.26)	1.43 ± 0.24 (0.95, 1.91)	2.17 ± 0.21 (1.75, 2.59)
Week 2	1.36 ± 0.29 (0.78, 1.93)	1.20 ± 0.28 (0.64, 1.76)	2.08 ± 0.25 (1.59, 2.56)
Week 4	1.23 ± 0.30 (0.65, 1.82)	1.10 ± 0.29 (0.52, 1.68)	1.91 ± 0.26 (1.39, 2.43)
Week 8	1.07 ± 0.27 (0.54, 1.59)	0.97 ± 0.27 (0.44, 1.50)	1.64 ± 0.24 (1.16, 2.12)
Week 12	1.12 ± 0.30 (0.53, 1.71)	1.00 ± 0.27 (0.46, 1.54)	1.77 ± 0.28 (1.22, 2.32)
Difference from the non-SJP group at 12 weeks	-0.65 ± 0.41 (-1.46, 0.16) *P* = 0.116	-0.77 ± 0.39 (-1.54, 0.01) *P* = 0.052	

Abbreviations: CI = confidence interval; LogMAR = logarithm of the minimum angle of resolution; SE = standard error.

Data are expressed as estimates ± SE (95% CI).

All estimates and *P* values were calculated using a general linear model with a compound symmetry working correlation matrix and robust variance estimators.
