# Supplementary material for: Safety and Efficacy of Orally Administered SJP-0008 in Central Retinal Artery Occlusion: A Phase IIa Randomized Clinical Trial
Source: Ophthalmol Sci. 2025 Oct 10;6(1):100965. doi: 10.1016/j.xops.2025.100965 (PMC12662090; doi:10.1016/j.xops.2025.100965)
Supplement: Table S3 [file mmc4.pdf]

Supplementary Table S3. Adverse Events by Planned Treatment.

		Planned treatment					
		SJP-0008, 100 mg (n = 10)			SJP-0008, 200 mg (n = 10)		
		Number of cases	Number of patients	%	Number of cases	Number of patients	%
**Body system or organ class**	**Dictionary-derived term**						
Cardiac disorders	Presyncope	0	0	0	1	1	10.0
	-- Subtotal --	0	0	0	1	1	10.0
Eye disorders	Allergic conjunctivitis	1	1	10.0	0	0	0
	Optic disc hemorrhage	1	1	10.0	1	1	10.0

		Planned treatment					
		SJP-0008, 100 mg (n = 10)			SJP-0008, 200 mg (n = 10)		
		**Number of cases**	**Number of patients**	**%**	**Number of cases**	**Number of patients**	**%**
	Retinal neovascularization	0	0	0	1	1	10.0
	Retinal vascular disorder	1	1	10.0	0	0	0
	Vitreous hemorrhage	1	1	10.0	0	0	0
	-- Subtotal --	4	4	40.0	2	2	20.0
Gastrointestinal disorders	Diarrhea	0	0	0	1	1	10.0
	Stomatitis	0	0	0	1	1	10.0
	Toothache	0	0	0	1	1	10.0

		Planned treatment					
		**SJP-0008, 100 mg (n = 10)**			**SJP-0008, 200 mg (n = 10)**		
		**Number of cases**	**Number of patients**	**%**	**Number of cases**	**Number of patients**	**%**
	-- Subtotal --	0	0	0	3	2	20.0
Injury, poisoning, and procedural complications	Post-procedural pruritus	0	0	0	1	1	10.0
	-- Subtotal --	0	0	0	1	1	10.0
Investigations	Blood creatine phosphokinase increased	2	2	20.0	0	0	0
	C-telopeptide decreased	0	0	0	2	2	20.0
	C-telopeptide increased	0	0	0	1	1	10.0

		Planned treatment					
		**SJP-0008, 100 mg (n = 10)**			**SJP-0008, 200 mg (n = 10)**		
		**Number of cases**	**Number of patients**	**%**	**Number of cases**	**Number of patients**	**%**
	Gamma-glutamyl transferase increased	0	0	0	1	1	10.0
	Platelet count decreased	1	1	10.0	0	0	0
	-- Subtotal --	3	2	20.0	4	4	40.0
Metabolism and nutrition disorders	Hypophosphatemia	0	0	0	2	2	20.0
	-- Subtotal --	0	0	0	2	2	20.0
	Back pain	1	1	10.0	0	0	0

		Planned treatment					
		SJP-0008, 100 mg (n = 10)			SJP-0008, 200 mg (n = 10)		
		**Number of cases**	**Number of patients**	**%**	**Number of cases**	**Number of patients**	**%**
Musculoskeletal and connective tissue disorders	-- Subtotal --	1	1	10.0	0	0	0
Nervous system disorders	Embolic cerebral infarction	1	1	10.0	0	0	0
	Hypoesthesia	0	0	0	1	1	10.0
	-- Subtotal --	1	1	10.0	1	1	10.0
Renal and urinary disorders	Renal impairment	1	1	10.0	0	0	0

		Planned treatment					
		SJP-0008, 100 mg (n = 10)			SJP-0008, 200 mg (n = 10)		
		**Number of cases**	**Number of patients**	**%**	**Number of cases**	**Number of patients**	**%**
	-- Subtotal --	1	1	10.0	0	0	0
Respiratory, thoracic, and mediastinal disorders	Cough	1	1	10.0	0	0	0
	Productive cough	1	1	10.0	0	0	0
	-- Subtotal --	2	1	10.0	0	0	0
Skin and subcutaneous tissue disorders	Pruritus	0	0	0	1	1	10.0
	-- Subtotal --	0	0	0	1	1	10.0

		Planned treatment					
		SJP-0008, 100 mg (n = 10)			SJP-0008, 200 mg (n = 10)		
		Number of cases	Number of patients	%	Number of cases	Number of patients	%
Vascular disorders	Embolic cerebral infarction	1	1	10.0	0	0	0
	Retinal hemorrhage	1	1	10.0	0	0	0
	Withdrawal hypertension	1	1	10.0	0	0	0
	-- Subtotal --	3	3	30.0	0	0	0
== Overall ==	-- Total --	15	8	80.0	15	7	70.0

A single patient could encounter multiple adverse events.
